# High frequency of Fredrickson's phenotypes IV and IIb in Brazilians infected by human immunodeficiency virus

**DOI:** 10.1186/1471-2334-5-47

**Published:** 2005-06-14

**Authors:** Edilma MV Albuquerque, Eliana C de Faria, Helena CF Oliveira, Daniela O Magro, Lucia N Castilho

**Affiliations:** 1 Departamento de Patologia Clinica, Faculdade de Ciências Médicas, Universidade Estadual de Campinas- UNICAMP- Campinas, SP, Brazil; 2 Núcleo de Medicina e Cirurgia Experimental, Faculdade de Ciências Médicas Universidade Estadual de Campinas- UNICAMP- Campinas, SP, Brazil; 3 Departamento de Fisiologia e Biofísica, Instituto de Biologia, Universidade Estadual de Campinas- UNICAMP- Campinas, SP, Brazil; 4 Departamento de Medicina Preventiva e Social, Faculdade de Ciências Médicas, Universidade Estadual de Campinas- UNICAMP- Campinas, SP, Brazil

## Abstract

**Background:**

Human immunodeficiency virus (HIV) infection is very prevalent in Brazil. HIV therapy has been recently associated with coronary heart disease (CHD). Dyslipidemia is a major risk factor for CHD that is frequently described in HIV positive patients, but very few studies have been conducted in Brazilian patients evaluating their lipid profiles.

**Methods:**

In the present work, we evaluated the frequency and severity of dyslipidemia in 257 Brazilian HIV positive patients. Two hundred and thirty-eight (93%) were submitted to antiretroviral therapy (224 treated with protease inhibitors plus nucleoside reverse transcriptase inhibitors, 14 treated only with the latter, 12 naive and 7 had no records of treatment).

The average time on drug treatment with antiretroviral therapy was 20 months. None of the patients was under lipid lowering drugs. Cholesterol, triglyceride, phospholipid and free fatty acids were determined by enzymatic colorimetric methods. Lipoprotein profile was estimated by the Friedewald formula and Fredrickson's phenotyping was obtained by serum electrophoresis on agarose. Apolipoprotein B and AI and lipoprotein "a" were measured by nephelometry.

**Results:**

The Fredrickson phenotypes were: type IIb (51%), IV (41%), IIa (7%). In addition one patient was type III and another type V. Thirty-three percent of all HIV+ patients presented serum cholesterol levels ≥ 200 mg/dL, 61% LDL-cholesterol ≥ 100 mg/dL, 65% HDL-cholesterol below 40 mg/dL, 46% triglycerides ≥ 150 mg/dL and 10% have all these parameters above the limits. Eighty-six percent of patients had cholesterol/HDL-cholesterol ratio ≥ 3.5, 22% increased lipoprotein "a", 79% increased free fatty acids and 9% increased phospholipids. The treatment with protease inhibitors plus nucleoside reverse transcriptase inhibitors increased the levels of cholesterol and triglycerides in these patients when compared with naïve patients. The HDL-cholesterol (p = 0.01) and apolipoprotein A1 (p = 0.02) levels were inversely correlated with the time of protease inhibitor therapy while total cholesterol levels had a trend to correlate with antiretroviral therapy (p = 0.09).

**Conclusion:**

The highly varied and prevalent types of dyslipidemia found in Brazilian HIV positive patients on antiretroviral therapies indicate the urgent need for their early diagnosis, the identification of the risk factors for CHD and, when needed, the prompt intervention on their lifestyle and/or with drug treatment.

## Background

The prognosis of patients with acquired immune deficiency syndrome (AIDS) was so limited until recently, that the medical interest in other long term health problems was irrelevant. The potency and sustained efficacy of the highly active antiretroviral therapy (HAART) for treating these patients brought a profound positive impact on their life expectancy reducing their mortality rates from AIDS [1].

Several reports [1-10] described the worsening of coronary heart disease (CHD) and vascular atherosclerotic complications in HIV+ patients after HAART therapy. Recently, the DAD study (Data Collection on Adverse Events of Anti-HIV Drugs) showed an increase in the risk of myocardial infarction (MI) from 0.30% in patients with no antiretroviral therapy to 1.07% in patients receiving these therapies, over a 3 year period [10].

Dyslipidemia is a major risk factor for the development of CHD. It has also been reported that the AIDS infection itself is capable of inducing dyslipidemia [11-14].

Hypertriglyceridemia was the first finding to be reported in HIV-infected patients, but other lipid abnormalities have also been described such as hypocholesterolemia, hypobetalipoproteinemia, hypoalphalipoproteinemia and, more rarely, hypercholesterolemia [11,13,15-17].

Brazil is the epicenter of the epidemic in South America and accounts for three-fifths of reported AIDS cases and 57% in Latin America and Caribbean. Among the population of high risk the prevalence is 42% [18].

Up to now two local studies [14,19] explored the dyslipidemia of Brazilian HIV+ patients but both in a small number of cases.

Therefore the aim of this study was to determine the prevalence and severity of different types of dyslipidemia in a large local HIV+ Brazilian population using antiretroviral therapy. Serum lipids, lipoproteins and apolipoproteins were measured and the effects on them of the viral load, CD4 counting and duration of therapy were evaluated.

## Methods

This study was approved by the Medical Ethics Committee of the Medical Sciences Faculty of the University of Campinas. Written consent was obtained from the patients or their relative for publication of study.

Two hundred and fifty seven HIV+ patients were enrolled in the protocol. They were attended in the Infectious Diseases Clinic at the University of Campinas. Sixty-two percent were men and 38% were women, with an average age of 35 ± 8 years, body weight average 67 ± 13 Kg and body mass indexes (BMI) 24 ± 4 Kg/m^2^. Two hundred and thirty-eight (93%) were submitted to antiretroviral therapy (224 treated with protease inhibitors plus nucleoside reverse transcriptase inhibitors, 14 treated only with the latter, 12 naïve and 7 had no records of treatment). The average time on drug treatment with protease inhibitors was 20 months (range 2 to 47 months). None of the patients was under lipid lowering drugs and any other disease was described in their records.

The measurements of fasting serum cholesterol (Chol), HDL-cholesterol (HDL-chol) and triglycerides (TG) were obtained by enzymatic colorimetric methods (automated Mega-Bayer system). The LDL-cholesterol (LDL-chol) and VLDL-cholesterol (VLDL-chol) were estimated by Friedewald's equation. In patients with triglyceride levels above 400 mg/dL (n = 16), Friedwald's equation was not used. The apolipoproteins A1 (Apo A1), B100 (Apo B) and lipoprotein "a" [Lp (a)] were measured by nephelometric methods (semi-automated Beckman Array-360 system) [20]. Free fatty acids (FFA) and phospholipids (PL) were determined by enzymatic colorimetric methods (Wako Chemicals GmbH, Japan). The plasma lipoproteins were fractioned by agarose gel electrophoresis (Paragon Electrophoresis System – Beckman, Ca, USA). The bands were carefully analyzed by visual inspection [21]. Apo E genotyping was performed by PCR assay [21] in 3 cases suspected of type III phenotype.

In a sub-group of patients presenting Chol and/or TG, respectively equal to or above 200 and 150 mg/dL (n = 141), the criterion for describing the dyslipidemia was in accord with WHO/ Fredrickson's classification [22].

The lipid profiles were analyzed using the values recommended by the National Cholesterol Education Program (NCEP, 2001) [23]. The desirable Chol/HDL-chol ratio was lower than 3.5 [24] and was used as an CHD risk index.

The reference intervals for apolipoproteins, Lp (a), PL and FFA were obtained from the manufacturers' recommendations. Apo A1: 94–178 mg/dL and 101–199 mg/dL; Apo B100: 52–109 mg/dL and 40–103 mg/dL for men and women, respectively; Lp(a) below 30 mg/dL; PL: 150–250 mg/dL and FFA: 0.1–0.6 mEq/L.

Immunologic evaluation included measurements of lymphocyte sub-populations, CD4 and CD8, by flow citometry and the determination of the viral load by PCR.

Kruskal-Wallis with post test Dunn was employed to compare the lipidic parameter averages between the 2 groups of antiretroviral therapy users and between the treated groups and naïve patients. Ancova and Pearson's tests were employed to correlate lipid profiles and CD4 cells, viral load and time of use of protease inhibitors. BMI, age and gender were chosen as covariates. Differences were considered significant when p ≤ 0.05.

## Results

The patients were classified according to the Center for Disease Control (CDC) -1993 [25] as follows: stage A, 39.3%; stage B, 12.4%; stage C, 45.6% (44.0% belonging to the clinical C3 category) and 2.7% were not clinically defined. There was an inversion of CD4 counting (average 337 cells/mm^3^) and CD8 counting (average 947 cells/mm^3^) that may be explained by the fact that approximately 44% of the patients belonged to the C3 category.

Forty-four percent of the patients who had their viral load measured presented less than 400 copies/mL, 29% between 400–10,0000 copies/mL and 27% above 10,000 copies/mL.

Fifty-five percent of total HIV+ population (141 patients) was selected by their high Chol and/or TG levels for further serum electrophoresis analysis. In eighteen patients the electrophoresis were not done and in 20 patients the results were inconclusive. The most frequent patterns according to Fredrickson's classification were type IV (41%) and IIb (51%) (Table 1). One patient was type III, another one type V and seven IIa.

**Table 1:** Percentual distribution of HIV+ patients according to WHO/Fredrickson classification in total HIV+ patients.

Type	Percent of dyslipidemic patients* (n = 103)	Percent of the total HIV+ population (n = 257)
IIa	7	3
IIb	51	20
III	1	0,4
IV	41	16
V	1	0,4

*Patients with chol and/or TG respectively, >200 and >150 mg/dL, were classified based on Fredrickson's classification

**Figure 1:**
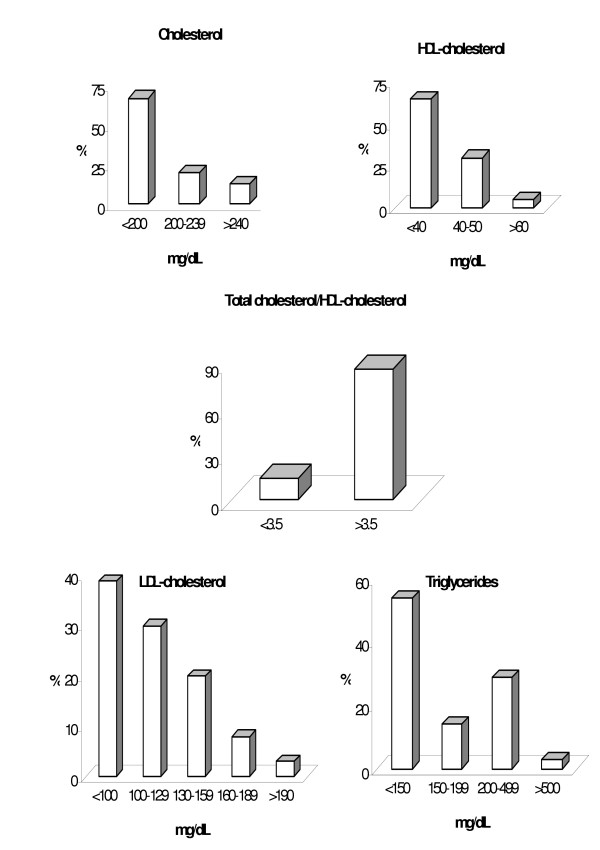
Lipid frequency distribution patterns in HIV+ patients (n = 236 to 257) according to NCEP's recommendations.

Patients' lipid, lipoprotein and apolipoprotein profiles are reported in Table 2. Figure 1 shows the percent distribution of HIV+ patients according to the NCEP (2001) classification [23]. Thirty-three percent of total HIV+ patients had Chol levels ≥ 200 mg/dL, 65% had HDL-chol below 40 mg/dL, 61% had LDL-chol ≥ 100 mg/dL; 46% had triglycerides ≥ 150 mg/dL and 86% had the cholesterol/HDL-chol ratio ≥ 3.5. Seventy-nine percent of the patients presented FFA values above the maximum reference value of 0.6 mEq/L. The average serum concentration of PL and Lp(a) were within the reference limits. However, 22% of the patients had Lp(a) above 30 mg/dL and 9% had PL above 250 mg/dL.

**Table 2:** Serum lipid, lipoprotein and apolipoprotein profiles in HIV+ patients (n = 257)

Variables^a^	Mean ± SD	Minimum	Maximum
Chol	185 ± 55	68	672
TG	192 ± 179	36	1512
HDL-chol	36 ± 12	12	73
LDL-chol*	112 ± 38	10	227
VLDL-chol*	31 ± 16	7	79
PL	192 ± 72	94	702
FFA	1.2 ± 0.75	0.10	6.3
Apo A1	121 ± 28	29	291
Apo B	88 ± 26	37	173
Lp(a)	21 ± 24	1	124
Chol/HDL-chol	5.6 ± 3	2	39.5

^a^Data expressed as mg/dL and FFA as mEq/L. * n = 241 patients

The mixed dyslipidemia in HIV+ patients are widely distributed and the frequency varied from 13% for TG or Chol + LDL-chol + HDL-chol to 38% for Chol+TG+HDL-chol.

The treatment with protease inhibitors plus nucleoside reverse transcriptase inhibitors increased significantly Chol and TG levels (p < 0.05) in these patients when compared to naïve HIV+ patients (187 ± 55 *vs *150 ± 21 mg/dL and 199 ± 187 *vs *106 ± 51 mg/dL for Chol and TG, respectively), no differences were found between the two drugs groups.

**Figure 2:**
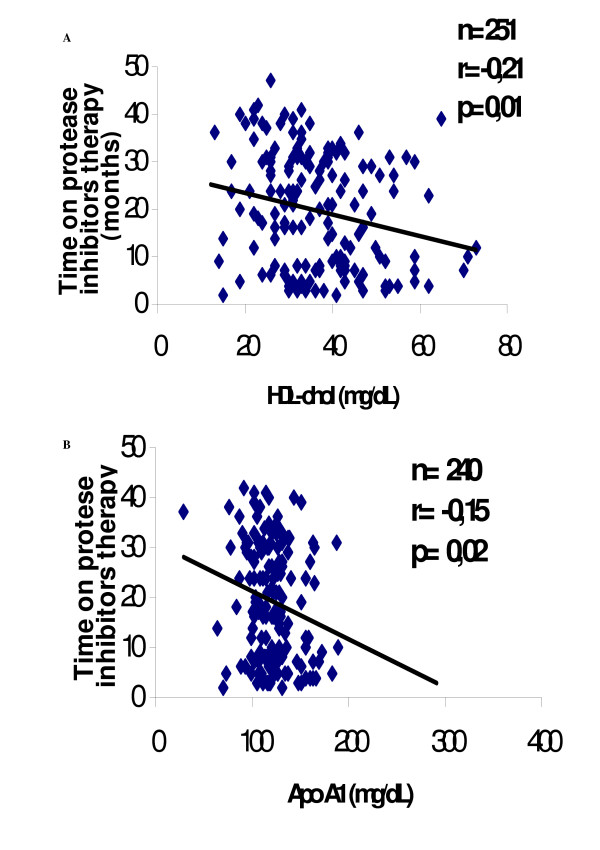
Correlations between time on protease inhibitor therapy and HDL-chol, (panel A) and Apo A 1 (panel B).

There was a significant negative correlation (Figure 2) between the time of protease inhibitors (PI) therapy and HDL-chol (r = -0.214) and Apo A1 (r = -0.154) levels (p = 0.01 and p = 0.02, respectively). Also a significant negative association (p = 0.03) was found between Chol levels and the viral load. Total cholesterol levels have a trend to be associate with protease inhibitor therapy (p = 0.09).

## Discussion

Dyslipidemia using the WHO/Fredrickson classification in a Brazilian HIV+ patients is examined here for the first time.

Schmidt et al [25] described dyslipidemia in 57% of 98 HIV+ individuals treated with protease inhibitors and found a different prevalence from our group: phenotypes IV and V were more frequent than IIb and IIa. Maus et al [26] just recently presented this classification in 187 treated German HIV+ individuals. Some curious differences were found between the phenotyping of the 2 populations: types IIb and IV was equally frequent in our study, but the German group type IV was the predominant one.

The data presented here are alarming. A very high prevalence of different types of dyslipidemia was found in the population of 257 HIV+ patients: 33% showed increased cholesterol, 46% increased triglycerides, decreased HDL-cholesterol level in 65%, cholesterol/HDL-chol ratio ≥ 3.5 in 86%, increased LDL-cholesterol in 61%, increased Lp(a) in 22%, increased free fatty acids in 79% and increased phospholipids in 9%. All of these profiles are strongly related to CHD risk.

In this study we showed that in patients using antiretroviral therapy there was an augment in the concentrations of plasma cholesterol and triglycerides when compared to naïve patients. We also found negative associations between the time on PI therapy and HDL-chol and Apo A1 levels and a trend to a positive correlation between the viral load and cholesterol levels.

Although HIV infection itself has been associated with altered lipid metabolism, substantial evidence indicates a role for some protease inhibitors and reverse transcriptase inhibitors in causing metabolic complications [27-32]. Many adverse reactions can be attributed to protease inhibitors, however; because several treatments use drug association regimens, it is difficult to find out the exact causal connection [33].

Several reports have associated hypertriglyceridemia with the use of antiretroviral therapy [6,8,30,33-37]; however, the increase in triglyceride levels was also described in HIV+ patients before the utilization of HAART [11,38]. Hypertriglyceridemia was highly prevalent in our and other studies [6,11-13,36,38-43]. This may also be secondary to cytokine production. Cytokines have been shown to increase hepatic lipid synthesis and/or decrease levels of lipoprotein lipase, which results in slower clearance of circulating triglyceride-rich particles [44]. Grunfeld et al [44] found a highly significant positive correlation between interferon-α and triglyceride levels. In our study, hypertriglyceridemia was present in 46% percent of the HIV+ patients. Thirty-six percent of the patient's hyperlipoproteinaemia was due to types IV and IIb (Table 3). The origin of the hypertrygliceridemia could be an increased hepatic VLDL-TG secretion rate, with high availability of FFA to the liver, secondary to insulin resistance, common in these patients [45,46]. Seventy nine percent of our patients presented high plasma levels of FFA, corroborating with this metabolic state.

We did not find significant correlations between triglycerides and viral load [16], CD4 levels [13,40] and the type of antiretroviral therapy [35], but we did find negative correlation between the duration of treatment with PI and HDL-chol (p = 0.01).

Low HDL-Chol was one of the major findings in this Brazilian HIV+ population (65%). These findings are in accordance with several authors [13,35,42]. Several metabolic processes may contribute to these low HDL levels: decrease in free cholesterol removal from cells, low cholesterol esterification rate and high cholesteryl ester transfer from HDL to apo-B containing lipoproteins [47].

Hypercholesterolemia was very prevalent in the dyslipidemic individuals described here: 58% are types IIa and IIb. Grunfeld et al.[44] didn't find significant differences in the cholesterol levels of patients affected by HIV: HIV+; HIV+ with AIDS or HIV negative, but Law et al. found raised cholesterol and triglyceride levels in patients receiving HAART, compared with patients not receiving HAART [10]. Our results are in agreement with Law's paper.

Grunfeld et al [39] showed a significant decrease in cholesterol levels in the HIV+ and HIV+ with AIDS patients, when compared to HIV- subjects. Shor-Posner et al [48] observed hypocholesterolemia (chol <150 mg/dL) in 41% of HIV+ and in 17% of HIV- patients. Christeff et al [40] reported decreased concentrations of cholesterol and phospholipids in HIV+ patients, except in those with low CD4 counting (400–150 cells/mm^3^). In this study, 29% of the patients presented hypocholesterolemia. We found an inverse correlation between plasma cholesterol levels and the viral load.

The observed high frequency of hypercholesterolemia and hyperbetalipoproteinemia in HIV+ Brazilian patients were never reported previously (Table 1, Figure 1). Also analyzing the various patterns of the combined hyperlipidemia the most prevalent were alterations in TG plus HDL-chol and LDL-chol plus HDL-chol, respectively 35 and 32% ; total cholesterol plus TG and HDL-chol in 38% and 11% of patients had four different lipidic parameters altered.

## Conclusion

The high frequency of phenotypes IIb and IV found in the Brazilian HIV positive patients and the severity of the disturbances such as: hypercholesterolemia up to 672 mg/dL, hyperbetalipoproteinemia up to 227 mg/dL, hypertriglyceridemia up to 1512 mg/dL and hypoalphalipoproteinemia below 13 mg/dL, indicate the urgent need for their early diagnosis, the identification of the presence of other risk factors for CHD and, when needed, the prompt intervention on their lifestyle and/or drug treatment [49,50].

## Competing interests

The author(s) declare that they have no competing interests.

## Authors' contributions

EMVA performed the biochemical analyses, data calculations and interpretation of results and helped to write the manuscript. ECF helped in the clinical trials and to write the manuscript. HCFO helped to write the manuscript. DOM participated in the clinical trial of HIV patients. LNC planned and coordinated the whole study, and helped to write the manuscript. All authors read and approved the final manuscript.

## Pre-publication history

The pre-publication history for this paper can be accessed here:


